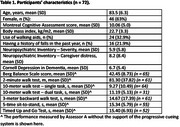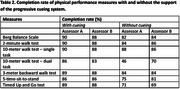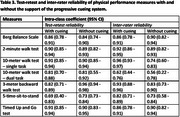# Improving the assessment of physical performance in older adults with dementia using a progressive cueing system

**DOI:** 10.1002/alz70858_102511

**Published:** 2025-12-26

**Authors:** Wayne LS Chan, Tamis Pin, Raymond Chung, Vincent C.T. Mok, Keith Hill

**Affiliations:** ^1^ The Hong Kong Polytechnic University, Hong Kong, Hong Kong, Hong Kong; ^2^ The Hong Kong Polytechnic Unviersity, Hong Kong, Hong Kong, Hong Kong; ^3^ Division of Neurology, Department of Medicine and Therapeutics, The Chinese University of Hong Kong, Hong Kong SAR, Hong Kong; ^4^ Rehabilitation, Ageing and Independent Living (RAIL) Research Centre, Frankston, VIC, Australia

## Abstract

**Background:**

Older adults with dementia have difficulties following and completing common standardized physical performance measures. Cues are shown to be potentially effective in enhancing their completion rate of certain physical performance measures (e.g., walk tests). Nevertheless, the effects of cues on the completion rate, reliability, and validity of common physical performance measures in this population have not been thoroughly investigated.

This study aims to evaluate the effects of a progressive cueing system on the completion rate, test‐retest reliability, and inter‐rater reliability of physical performance measures in older adults with dementia.

**Method:**

Older adults with dementia who can walk 10 m with or without a walking aid independently were recruited. The participants completed the Berg Balance Scale, the 2‐minute walk test, the 10‐meter walk test – single task and dual task, the 3‐meter backward walk test, and the 5‐time sit‐to‐stand test with or without the support of the progressive cueing system. The progressive cueing system consists of five levels: (0) no cue, (1) verbal cue, (2) modeling, (3) one‐off physical cue, and (4) repeated physical cues. Cues were provided if the participants failed to follow any procedures of the measures (e.g., a sudden discontinuation of movement due to being distracted by the surroundings). Two assessors conducted the physical performance measures on six separate testing occasions within three weeks. The mean percentages of the physical performance measures completed by the participants and the intra‐class correlation coefficients (ICC) of the physical performance measures were evaluated.

**Result:**

Seventy‐two older adults with dementia were recruited (Table 1). The participants’ completion rate (with cueing = 83 – 90%; without cueing = 46 – 88%), test‐retest reliability (with cueing ICC = 0.69 – 0.91; without cueing ICC = 0.73 – 0.96), and inter‐rater reliability (with cueing = 0.62 – 0.96; without cueing = 0.56 – 0.90) of the physical performance measures were generally higher when the progressive cueing system supported the participants during the measurement (Tables 2 and 3).

**Conclusion:**

Our findings show that the progressive cueing system is potentially effective in improving the completion rate, test‐retest, and inter‐rater reliability of physical performance measures in older adults with dementia.